# Exercise and Circadian Rhythm Regulation: Mechanisms, Resilience, and Therapeutic Potential for Neurological Diseases

**DOI:** 10.1002/cns.71035

**Published:** 2026-08-17

**Authors:** Munehiro Demura, Penelope Gregory, Norito Fukuda, Eng H. Lo, Ken Arai

**Affiliations:** ^1^ Neuroprotection Research Laboratory, Department of Radiology Massachusetts General Hospital and Harvard Medical School Charlestown Massachusetts USA; ^2^ Neuroprotection Research Laboratory, Department of Neurology Massachusetts General Hospital and Harvard Medical School Charlestown Massachusetts USA; ^3^ Department of Neurosurgery, Graduate School of Medicine, Faculty of Medicine University of Yamanashi Yamanashi Japan

**Keywords:** Alzheimer's disease, circadian rhythms, exercise, neurological diseases, non‐photic zeitgeber, stroke

## Abstract

**Background:**

Circadian rhythms regulate sleep–wake cycles, hormonal secretion, metabolism, and immune responses. Disruption of these rhythms is linked to the onset and progression of various neurological diseases. Emerging evidence suggests that exercise, a non‐photic zeitgeber, can modulate circadian function at both central and peripheral levels by regulating core clock gene expression, synchronizing hormonal and metabolic rhythms, and reducing neuroinflammation. Through these mechanisms, exercise enhances the amplitude, stability, and phase alignment of circadian rhythms and may promote physiological resilience.

**Review:**

This review summarizes the molecular and systemic mechanisms through which exercise supports circadian homeostasis and discusses their relevance to stroke and Alzheimer's disease. In both conditions, circadian misalignment contributes to pathological processes including oxidative stress, neuroinflammation, and impaired neurovascular repair. By restoring circadian alignment, appropriately timed exercise may improve neurological outcomes through effects on neuroplasticity, immune regulation, and metabolic synchronization.

**Summary:**

Taken together, current evidence may suggest that exercise can be a promising non‐pharmacological strategy to enhance circadian resilience, reduce disease vulnerability, and support neurological recovery.

## Graphical abstract

Exercise acts as a non‐photic zeitgeber that enhances circadian rhythm alignment, reduces neuroinflammation and oxidative stress, and supports neuroprotection in CNS diseases, including stroke and Alzheimer's disease.

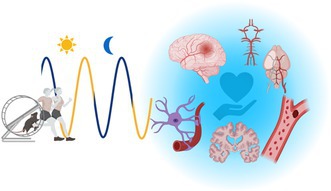

## 1 Introduction

Circadian rhythms are essential for regulating a wide range of physiological and behavioral processes in mammals, operating on a 24‐h cycle. These rhythms are primarily controlled by the suprachiasmatic nucleus (SCN), located in the hypothalamus, which serves as the central pacemaker for the body's internal clocks. The SCN synchronizes peripheral oscillators throughout the body, governing vital functions such as the sleep–wake cycle, hormonal secretion, and body temperature [1, 2, 3]. While the SCN maintains its function through intrinsic mechanisms, the rhythm is also influenced by external cues, known as zeitgebers, including light exposure, dietary patterns, physical activity, and environmental temperature [4, 5]. Light exposure is considered the most potent zeitgeber, directly influencing the SCN and helping align the circadian system with the external light–dark cycle [6, 7, 8]. However, non‐photic cues, such as physical activity, diet, and temperature, have also emerged as powerful regulators of circadian rhythm. Exercise has shown significant potential for modulating the circadian clock, helping to restore synchrony when circadian rhythms become misaligned, particularly in cases involving shift work or jet lag [3, 9, 10, 11]. Emerging evidence also suggests a bidirectional relationship between circadian rhythms and neurological diseases, with disruptions in circadian rhythms contributing to both the onset and progression of several neurological diseases [12].

Several thoughtful reviews have addressed exercise as a non‐photic zeitgeber and examined its role in regulating skeletal muscle clocks, metabolic homeostasis, exercise timing, shift work adaptation, and metabolic disease [9, 13]. Nevertheless, the relationship between exercise, circadian rhythms, and neurological diseases has received little systematic attention. Increasing evidence suggests that circadian disruption is involved in both the development and progression of neurological diseases. Altered circadian rhythms may contribute to neuroinflammation, oxidative stress, metabolic dysfunction, and impaired tissue repair, which can worsen disease pathology and clinical outcomes [12, 14, 15, 16]. Therefore, strategies that improve circadian alignment may have therapeutic potential for neurological disorders. Exercise may be one such strategy. In addition to its well‐established effects on cardiovascular and metabolic health, exercise can influence circadian regulation through multiple pathways. In fact, exercise has been shown to affect the expression of clock genes, synchronize central and peripheral oscillators, and regulate hormonal and metabolic rhythms. These effects may improve the stability and robustness of circadian rhythms and help maintain physiological homeostasis under conditions of stress or disease [17, 18, 19]. Furthermore, exercise‐induced changes in immune, endocrine, and metabolic signaling may also contribute to communication between peripheral tissues and the central circadian system.

**FIGURE 1:**
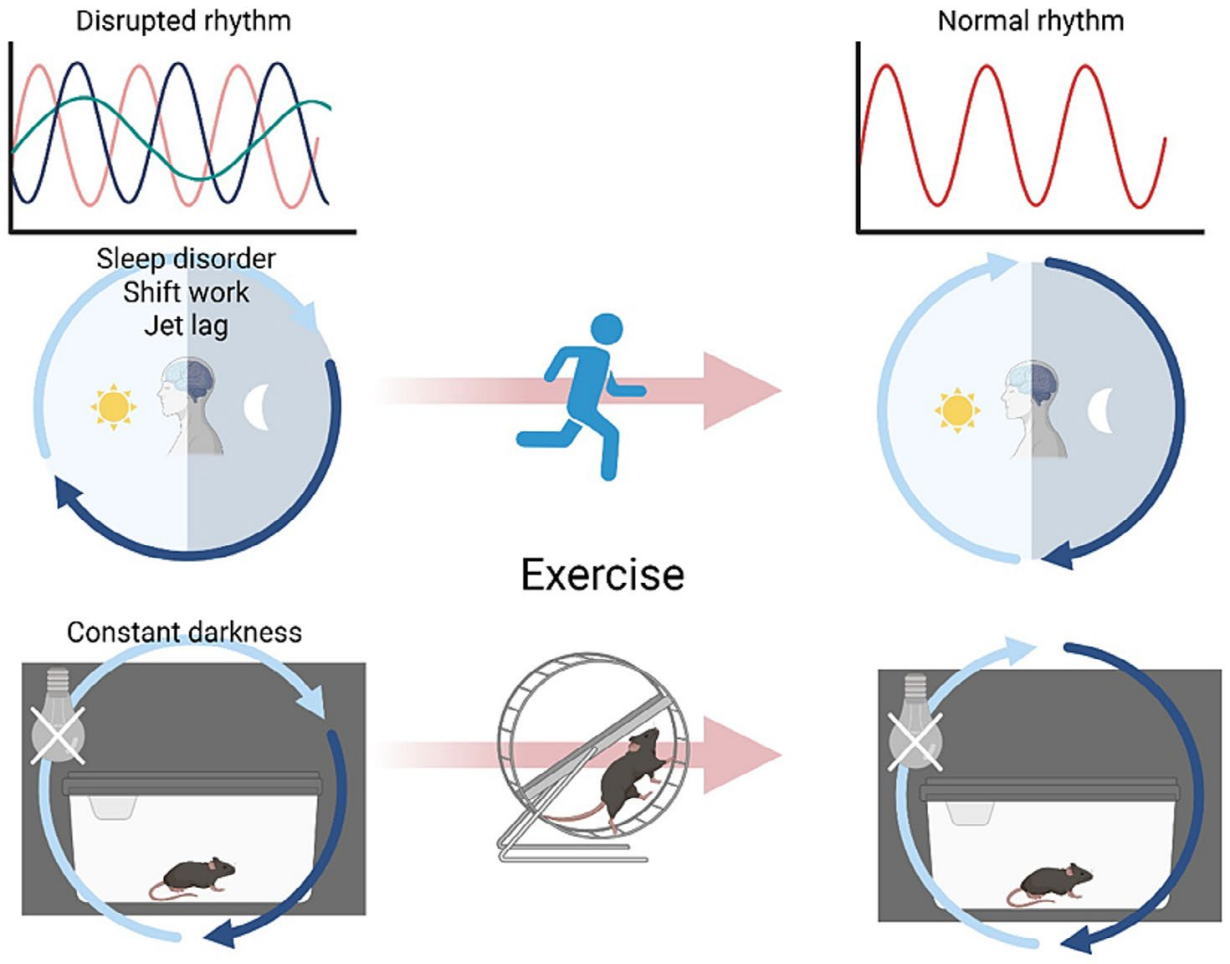
Exercise‐induced resynchronization of disrupted circadian rhythms in humans and rodents. Circadian rhythms can be disrupted by various environmental and behavioral factors, such as shift work, jet lag, and sleep disorders in humans, or constant darkness conditions in rodents. These disruptions lead to misalignment between the central clock in the suprachiasmatic nucleus (SCN) and peripheral oscillators, contributing to physiological and cognitive dysfunction. Exercise serves as a potent non‐photic zeitgeber that promotes circadian entrainment. In humans, timed physical activity helps restore normal sleep–wake cycles and hormonal rhythms. In rodent models, scheduled voluntary exercise in constant darkness conditions restores behavioral and molecular rhythmicity, even in the absence of light cues. Together, these findings highlight the role of exercise in resynchronizing disrupted circadian rhythms across species.

In this review, we aim to summarize current evidence regarding the effects of exercise on circadian rhythms and discuss the potential implications for neurological diseases. We focus on the molecular and physiological mechanisms through which exercise influences circadian regulation and examine how these mechanisms may contribute to disease modification in stroke and Alzheimer's disease (AD) (Figure 1), partly because these two conditions provide useful examples of circadian dysfunction. In stroke, circadian disturbances occur following acute brain injury and are associated with neuroinflammation, vascular dysfunction, and impaired recovery. In contrast, AD is characterized by progressive disruption of sleep–wake rhythms, neurodegeneration, and metabolic abnormalities [14, 15, 16]. Although the underlying mechanisms differ, circadian dysregulation would contribute to disease progression in both conditions. Finally, we discuss the concept of circadian resilience and highlight the potential of appropriately timed exercise as a strategy to maintain circadian homeostasis, reduce disease vulnerability, and support neurological recovery.

## 2 Exercise‐Induced Remodeling of Circadian Rhythms

**FIGURE 2:**
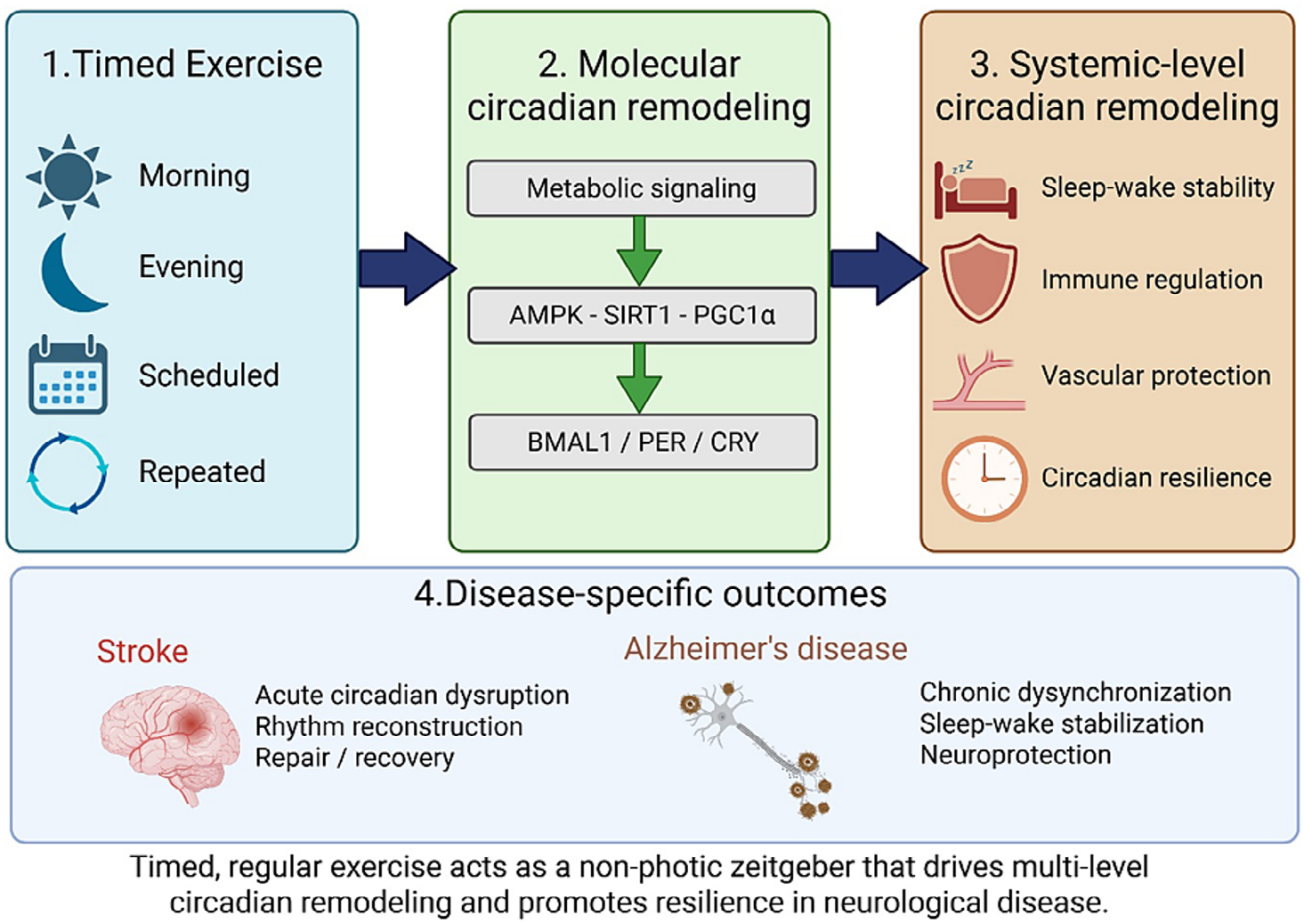
Mechanistic framework of exercise‐induced circadian remodeling in neurological disease. Timed and repeated exercise acts as a non‐photic zeitgeber that induces multi‐level circadian remodeling through metabolic and clock‐related signaling pathways. Exercise‐associated metabolic signaling, including AMPK–SIRT1–PGC‐1α pathways, interacts with core clock machinery (BMAL1, PER, and CRY) to influence systems‐level circadian regulation. These effects may contribute to sleep–wake stability, immune regulation, neurovascular protection, and circadian resilience. The downstream impact of exercise‐induced circadian remodeling may differ according to disease context. In stroke, exercise may support rhythm reconstruction and recovery after acute circadian disruption, whereas in Alzheimer's disease, exercise may promote sleep–wake stabilization and neuroprotection in the setting of chronic circadian desynchronization.

Exercise‐induced circadian remodeling should be understood as an active biological adaptation rather than a simple entrainment effect. Repeated exercise bouts generate rhythmic energetic stress, redox signaling, mitochondrial remodeling, neuroendocrine activation, and transient immune perturbation, all of which influence central and peripheral clock systems. Mechanistically, pathways such as AMPK‐SIRT1‐BMAL1 signaling, PGC‐1α‐dependent mitochondrial regulation, exercise‐induced epigenetic remodeling, and immune‐clock coupling provide plausible links between physical activity and enhanced circadian amplitude, stability, and synchronization [20]. This framework helps explain why regular and appropriately timed exercise may ameliorate circadian disruption and promote physiological resilience in neurological diseases. Notably, exercise timing also plays an important role in circadian remodeling. Morning and evening exercise exert distinct phase‐shifting effects on central and peripheral oscillators, contributing to long‐term circadian plasticity (Figure 2). Evidence from rodent and human studies indicates that repeated activation of metabolic, hormonal, and immune pathways reinforces coupling between the SCN and peripheral clocks, establishing a more resilient circadian network that resists environmental and pathological perturbations [13, 21, 22, 23, 24, 25, 26, 27, 28, 29, 30].

### 2.1 Amplitude Enhancement and Systemic Synchronization

One of the most prominent effects of long‐term exercise is the enhancement of rhythm amplitude across multiple biological levels. Regular physical activity increases the oscillatory range of core clock gene expression, including Bmal1, Per2, and Cry1, in skeletal muscle, liver, and vascular tissues, leading to stronger rhythmicity [31, 32, 33]. This amplification is thought to involve metabolic signaling pathways, including AMPK‐ and SIRT1‐mediated regulation of BMAL1 signaling and PGC‐1α‐dependent mitochondrial regulation, which translate repeated energetic stress into reinforcement of peripheral clock machinery [27, 28, 29, 30, 34]. At the systemic level, exercise promotes coupling among peripheral clocks by synchronizing endocrine and autonomic outputs with SCN activity [35]. Circulating hormones such as cortisol, insulin, and adiponectin exhibit greater rhythmic amplitude following chronic exercise training, reflecting strengthened hypothalamic–pituitary‐peripheral feedback [36]. In addition, rhythmic secretion of myokines, including interleukin‐6, irisin, and BDNF, links muscle activity to central and peripheral timing networks [37]. These coordinated responses enhance circadian coherence and support optimal energy distribution, vascular function, and neuronal excitability throughout the 24‐h cycle.

### 2.2 Stability and Resilience Against Stress

Beyond enhancing circadian amplitude, regular exercise may also improve the resilience of the circadian system. Exercise stabilizes oscillatory cycles under challenging conditions, such as jet lag, sleep deprivation, and chronic stress [35, 38]. Experimental models demonstrate that pre‐training with voluntary or forced exercise accelerates re‐entrainment following light‐shift paradigms and mitigates internal desynchronization between the SCN and peripheral organs [39, 40]. Mechanistically, this stabilization involves stress‐responsive pathways such as AMPK, SIRT1, and HSP70, which maintain redox balance and preserve cellular timekeeping under oxidative stress [14, 34]. Exercise‐induced increases in NAD^+^ availability support SIRT1‐mediated deacetylation of BMAL1 and PER2, enhancing precision in transcriptional feedback and buffering the clock against metabolic noise [41]. These molecular adaptations contribute to the ability of the circadian system to maintain temporal integrity despite environmental or physiological stressors. Clinically, enhanced rhythmic stability associated with habitual exercise correlates with improved sleep efficiency, reduced cortisol reactivity, and greater mood stability, suggesting that circadian resilience contributes to many psychological and metabolic benefits of physical activity [42].

### 2.3 Integration of Molecular, Metabolic, and Immune Synchronization

Exercise serves as an integrative force that aligns molecular and systemic oscillations across metabolic and immune domains. Metabolomic analyses show that repeated exercise entrains rhythmic fluxes in glucose, lipid, and amino acid metabolism, promoting temporal organization of metabolic processes [43]. Activation of AMPK and PGC‐1α signaling coordinates mitochondrial biogenesis and aligns cellular energy states with transcriptional oscillations of the core clock [44]. In parallel, exercise exerts substantial effects on immune rhythmicity. Regular training enhances diurnal variation in leukocyte trafficking, cytokine release, and microglial activity through glucocorticoid and sympathetic rhythms [45]. These immune oscillations influence central clock timing and neuroinflammation, forming a self‐reinforcing loop between physical activity and circadian balance [20].

In summary, chronic exercise transforms the circadian system into a high‐amplitude, stress‐resistant, and metabolically coherent network that supports optimal physiological function [37, 46]. This remodeling extends beyond simple entrainment and represents a durable adaptation that enhances the body's ability to anticipate, respond to, and recover from environmental and internal perturbations. These circadian adaptations are relevant in neurological diseases, where circadian disruption is common and clinically significant. Understanding how exercise‐induced remodeling interacts with disease‐related circadian disturbances provides insight into its therapeutic potential. Key operational parameters relevant to circadian‐targeted exercise interventions are summarized in Table 1.

**TABLE 1:** Operational parameters of circadian‐targeted exercise interventions. This table summarizes key exercise parameters relevant to circadian entrainment and neurological translation. Evidence is organized across human phase‐shifting studies, rodent scheduled‐exercise studies, and clinical neurological intervention studies because direct trials defining optimal circadian‐targeted exercise prescriptions in neurological disease remain limited.

Parameter	Evidence type	Circadian relevance	Translational consideration
Timing/chronotype	Human phase‐shifting studies [22, 23, 47, 48, 49, 50]	Exercise‐induced phase shifts depend on timing relative to circadian phase, melatonin rhythm, and chronotype.	Exercise timing should consider chronotype, habitual sleep schedule, endogenous circadian phase, and disease stage.
Regularity/fixed schedule	Rodent scheduled‐exercise studies [21, 26]	Repeated scheduled exercise can entrain behavioral rhythms and reorganize SCN/peripheral clock rhythms.	Fixed time‐of‐day exercise may function as a stronger zeitgeber than irregular activity.
Frequency/dose	Human and rodent evidence [21, 26, 48, 50]	Repeated bouts and sufficient daily exposure appear important for durable circadian adaptation.	Prescriptions should specify session duration, frequency, and adherence targets.
Intensity	Human phase‐shifting studies [23, 48, 49]	High‐, moderate‐, and low‐intensity exercise can influence circadian phase, but optimal intensity remains unclear.	Moderate or low‐intensity exercise may be more feasible in older adults and neurological patients.
Modality	Rodent and clinical evidence [21, 23, 26, 48, 49, 50]	Wheel running, cycling, stairclimber exercise, and walking can act as non‐photic circadian cues in different contexts.	Modality should be adapted to functional capacity, frailty, caregiver support, and rehabilitation goals.
Disease stage/clinical context	Neurological and translational studies [17, 18, 19, 50, 51]	Acute injury and chronic neurodegeneration may require different exercise goals and safety considerations.	Stroke may prioritize rhythm reconstruction and neurovascular repair, whereas AD may prioritize sleep–wake stability and adherence.

## 3 Disruption of Circadian Rhythm and Therapeutic Potential by Exercise in Neurological Diseases

### 3.1 Stroke

The relationship between stroke and circadian rhythm has gained increasing attention in recent years. Circadian rhythms are disrupted after stroke, contributing to neurological deficits, impaired recovery, and worse functional outcomes [14, 41, 42]. Circadian misalignment can also exacerbate post‐stroke inflammation and oxidative stress, increasing ischemic damage and limiting recovery. In particular, circadian disruption promotes pro‐inflammatory responses while impairing anti‐inflammatory mechanisms [43]. Recent studies further indicate that circadian regulation of neutrophil function influences stroke outcome. Neutrophil activation and NET formation vary across the day and impair collateral perfusion, resulting in larger infarct volumes and worse neurological outcomes [52]. Circadian timing also shapes broader immune responses after stroke, including immune cell trafficking across the blood, spleen, and brain. Stroke occurring during the inactive phase is associated with enhanced infiltration of activated T cells and altered peripheral immune dynamics [53]. In a nationwide multicenter cohort of more than 17,000 patients, night‐onset ischemic strokes were associated with greater neurological severity, increased risk of early neurological deterioration, and worse functional outcomes than day‐onset strokes [54]. These observations suggest that interventions that improve circadian alignment, including timed exercise, may enhance recovery. Circadian timing may also contribute to the translational failure of neuroprotective therapies, as several interventions shown to be effective during the inactive phase in nocturnal rodents lose efficacy during the active phase, which more closely reflects the circadian context of daytime stroke patients [55].

Notably, recent advances have identified the circadian clock system as a potential therapeutic target for improving stroke prognosis. Circadian regulation influences pathways involved in neuroprotection, immune balance, tissue repair, inflammation, neurogenesis, and vascular integrity [14, 41, 44, 45]. In this context, exercise, as a potent non‐photic zeitgeber, may represent a practical strategy to improve circadian alignment and support recovery after stroke [17, 18, 46, 51]. However, the extent to which the beneficial effects of exercise are mediated specifically through circadian clock modulation remains unclear. Targeted exercise interventions designed to optimize circadian alignment may therefore offer a novel approach to improve functional recovery and long‐term outcomes. But importantly, circadian clock modulation should not be viewed as uniformly protective. Although BMAL1 signaling has been linked to vascular integrity, metabolic homeostasis, and anti‐inflammatory regulation, its effects appear to depend on injury stage, cell type, circadian phase, and mode of clock manipulation [56, 57]. These findings suggest that exercise‐induced circadian remodeling is a context‐dependent process rather than a simple enhancement of clock activity. Future studies should clarify when and under what conditions exercise‐mediated clock modulation is beneficial.

#### 3.1.1 Ischemic Stroke

Ischemic stroke, the most common stroke subtype, results from cerebral blood flow obstruction caused by thrombus or embolus formation. Stroke incidence peaks during the early morning hours, coinciding with increases in blood pressure, sympathetic activity, and platelet aggregation [14, 58, 59].

Melatonin, a critical circadian regulator with neuroprotective properties, is frequently disrupted in ischemic stroke patients. A delayed dim light melatonin onset (DLMO) is commonly observed and is associated with poorer functional outcomes, likely due to the hormone's role in mitigating oxidative stress and inflammation [60]. Exercise has shown potential in restoring melatonin rhythms, particularly when performed in the morning, leading to advanced melatonin onset and improved neurovascular recovery [22, 47]. In addition, exercise under controlled dim light conditions has been demonstrated to facilitate phase shifts in melatonin rhythms, specifically inducing phase delays. Repeated bouts of moderate‐intensity exercise have been shown to significantly delay the DLMO compared to non‐exercising controls, emphasizing the importance of exercise timing relative to the endogenous circadian phase for optimizing phase‐shifting effects [48]. Similarly, a single session of low‐intensity nocturnal exercise under dim light conditions has been found to induce significant phase delays in the DLMO in both young and older adults. These findings indicate that exercise, even at lower intensities, can effectively shift circadian rhythms and that the phase‐shifting effects are preserved across different age groups [49].

Molecular disruptions of circadian clock genes, including BMAL1 and PER2, are also implicated in ischemic stroke pathology. These genes regulate key processes such as angiogenesis, neuronal survival, and inflammatory responses, all of which are crucial for post‐stroke recovery [41]. Consistent with these general mechanisms, previous studies have demonstrated that dysregulated immune responses, including excessive production of pro‐inflammatory cytokines such as IL‐6 and TNF‐α, contribute to secondary brain injury following ischemic stroke [43, 61]. Importantly, exercise has been demonstrated to restore immune balance by upregulating clock genes like BMAL1 and PER2 in immune cells, reducing pro‐inflammatory cytokine production, and enhancing anti‐inflammatory pathways. Exercise can align immune cell activity, such as macrophage migration and cytokine secretion, with the circadian system, helping to promote optimal timing for inflammation resolution and tissue repair. Hypoxia‐inducible factor‐1 (HIF‐1), which is activated during exercise, further supports recovery by promoting angiogenesis and reducing oxidative stress, thereby mitigating secondary damage [18]. By targeting circadian dysfunctions, exercise may offer a promising, non‐pharmacological intervention to enhance recovery and reduce the long‐term complications associated with ischemic stroke.

#### 3.1.2 Intracerebral Hemorrhage

Intracerebral hemorrhage (ICH) has also been linked to circadian rhythm disturbances. Emerging evidence suggests that disruption of circadian clock mechanisms may contribute to secondary brain injury following ICH through the dysregulation of inflammatory and metabolic pathways. Molecular components of the circadian clock, including BMAL1, CLOCK, PER, and CRY, are increasingly recognized as important modulators of post‐hemorrhagic brain injury. Experimental ICH models have demonstrated a significant reduction in BMAL1 expression, whereas BMAL1 overexpression suppresses oxidative stress and inflammatory responses, thereby reducing brain edema and neurological deficits [62]. Conversely, BMAL1 deficiency has been associated with enhanced NF‐κB activation and increased production of pro‐inflammatory cytokines, potentially amplifying neuroinflammation under conditions of circadian disruption [63]. These findings suggest that disrupted circadian rhythm‐related molecules may represent a novel pathological mechanism contributing to post‐ICH brain injury, and that targeting BMAL1 and other clock genes could offer a potential therapeutic strategy for ICH.

As discussed, exercise therapy could provide neuroprotection after ICH by helping regulate circadian rhythms, and this mechanism could suppress neuroinflammation and promote vascular repair. Physical activity upregulates circadian clock gene expression in peripheral tissues, including increases in BMAL1 and PER2 expression in skeletal muscle following exercise [63], and regular exercise also improves circadian synchronization, stabilizing physiological fluctuations such as immune responses and vascular tone. Epidemiological studies further suggest that habitual physical activity positively influences ICH outcomes. A prospective study found that patients with higher levels of pre‐stroke physical activity had less severe neurological deficits at the time of ICH onset and significantly lower 30‐day mortality rates compared to sedentary individuals. Specifically, the odds of presenting with mild neurological deficits were approximately 3.6 times higher, and the one‐month mortality rate was reduced by 70% in physically active patients [64]. Also in animal models of ICH, rehabilitation exercise introduced at an appropriate time promotes neuroregeneration, facilitating the recruitment of circulating stem cells and progenitor cells to the injured brain. This process supports neurogenesis and angiogenesis around the lesion site, accompanied by an increase in BDNF expression [65]. In animal experiments, ICH models that began rehabilitation within 1 week of onset exhibited superior motor function recovery compared to those who started rehabilitation after 2.5 weeks [66, 67]. In these early intervention groups, neuronal activation and expression of plasticity‐related markers were significantly enhanced [68]. Although the precise mechanisms by which exercise exerts its neuroprotective effects through circadian‐related signaling are not fully understood, exercise‐based interventions that target the molecular mechanisms of circadian rhythms may offer a promising approach to reducing secondary brain damage and improving functional recovery after ICH.

#### 3.1.3 Subarachnoid Hemorrhage

Similar to ischemic stroke and ICH, subarachnoid hemorrhage (SAH) is associated with circadian disruption. Aneurysmal SAH also exhibits a characteristic circadian pattern of onset, with peaks in the morning and evening [69]. Severe brain injury can affect the SCN, leading to alterations in hormone secretion and autonomic nervous system function. Disturbances in melatonin secretion rhythms have been reported, with loss of the normal day‐night pattern in some patients, suggesting dysfunction of the SCN and hypothalamic‐pineal axis [70]. In addition, systemic immune and inflammatory responses become activated after SAH, potentially disrupting normal circadian oscillations of cytokine secretion. Persistent elevation of pro‐inflammatory cytokines such as IL‐6 and IL‐1β during the acute phase also impairs circadian regulation [71]. These alterations in melatonin and cortisol rhythms, body temperature regulation, and inflammatory activity may exacerbate secondary brain injury and systemic complications following SAH.

The role of circadian clock gene expression in SAH has gained attention. Recent studies suggest that the timing of SAH onset may influence the severity of brain injury. In mouse models, SAH induced during periods of high clock gene expression resulted in less severe damage, whereas SAH occurring during low‐expression periods led to worsened outcomes. This indicates that circadian clock genes contribute to post‐hemorrhagic homeostasis, and their disruption may weaken brain resilience. In SAH patients, circadian gene dysregulation has been observed in cerebrospinal fluid, with Per2 levels increasing acutely, while peripheral blood expression remained unchanged. In addition, heme oxygenase‐1 (HO‐1) and carbon monoxide (CO) appear to regulate circadian genes after SAH. HO‐1 deficiency reduces PER1, PER2, and NPAS2 expression, worsening brain injury, whereas CO restores their oscillations and provides neuroprotection [72]. These findings suggest that circadian disruption may impact early brain injury and delayed cerebral vasospasm, highlighting its potential as a therapeutic target.

Exercise therapy is also being investigated as a potential neuroprotective strategy following SAH. Although intensive physical activity is not feasible during the acute phase, rehabilitation and moderate exercise during recovery may help resynchronize disrupted circadian rhythms and improve neurological outcomes. Exercise may normalize melatonin secretion, improve sleep quality, stabilize HPA axis function, and promote restoration of cortisol rhythmicity [16]. Early rehabilitation has also been associated with fewer complications and better functional outcomes in SAH patients, suggesting that physical activity may stabilize systemic homeostasis and promote neuroplasticity [73, 74]. Furthermore, exercise may enhance antioxidant enzyme activity and endothelial function, potentially reducing post‐SAH cerebral vasospasm and blood–brain barrier dysfunction. Aerobic exercise, for instance, has been proposed to modulate the circadian rhythm of vasoactive substances, such as the diurnal fluctuation of nitric oxide release, which could aid in vascular repair and homeostasis following SAH [63]. Although clinical evidence remains limited, early mobilization and rehabilitation appear to be safe and beneficial [75]. Future research should explore whether combining exercise therapy with circadian rhythm modulation strategies could further improve neurological prognosis after SAH.

### 3.2 Alzheimer's Disease

In contrast to stroke, which is characterized by acute circadian disruption after brain injury, Alzheimer's disease (AD) involves a chronic and progressive loss of circadian organization. Circadian dysfunction in AD is associated with sleep–wake fragmentation, impaired synchronization among brain regions, metabolic abnormalities, and persistent neuroimmune activation. These alterations may contribute to disease progression and cognitive decline, suggesting that interventions that strengthen circadian regulation, including exercise, may have therapeutic potential [14, 15, 16, 19, 41].

Clinically, AD is frequently accompanied by circadian rhythm disturbances, including fragmented sleep–wake cycles and “sundowning” behavioral agitation. Studies estimate that roughly 25%–66% of AD patients experience significant sleep disruption. These issues often precede the clinical diagnosis, suggesting that circadian dysfunction is an early event in AD [15, 76, 77]. At the molecular level, AD pathologies directly disrupt the circadian clock. Amyloid‐β promotes degradation of the core clock protein BMAL1, while tau accumulation in the SCN is associated with altered expression of clock genes such as PER2 and BMAL1 [16, 78]. These changes contribute to weakened hormonal rhythms, including reduced nocturnal melatonin secretion, and further impair sleep–wake regulation [79, 80]. Inflammation and metabolic dysfunction in AD further compound these effects, as chronic neuroinflammation can suppress BMAL1 in glial cells, while BMAL1 deficiency in the brain exacerbates oxidative damage. This indicates that clock disruption may worsen mitochondrial stress and increase neuronal vulnerability [16].

Exercise has been proposed as a neuroprotective intervention in AD, based largely on preclinical evidence, with emerging support from clinical studies. In transgenic AD mouse models, regular exercise reduces Aβ burden and preserves cognitive function through multiple mechanisms. Exercise modulates neuroinflammation by shifting microglia toward an anti‐inflammatory state, reducing IL‐1β and TNF‐α while increasing IL‐10 in the hippocampus. It also attenuates oxidative stress, as evidenced by reduced lipid peroxidation and increased antioxidant enzyme activity [81]. One key pathway involves SIRT1, a longevity‐associated deacetylase and clock regulator. Treadmill exercise in APP/PS1 mice activates SIRT1, promoting non‐amyloidogenic APP processing and reducing Aβ production [82]. Exercise also upregulates neuroprotective factors such as BDNF and downstream signaling pathways including CREB and PI3K‐Akt, which support synaptic plasticity and memory [83, 84, 85]. Beyond these disease‐specific effects, exercise acts as a non‐photic zeitgeber that strengthens circadian rhythms. Increased daily activity tends to normalize sleep–wake cycles in AD patients. Observational studies have shown that individuals with dementia who walk more exhibit better sleep quality and less sundowning agitation. Controlled trials further demonstrate that moderate exercise reduces nighttime awakenings and prolongs sleep duration [19, 50]. By improving sleep continuity and melatonin rhythms, exercise may further enhance overnight glymphatic clearance of brain toxins [86, 87, 88].

In summary, exercise intervention in AD addresses multiple pathological facets by reducing amyloid and inflammation, improving mitochondrial antioxidant defenses, and realigning circadian oscillators. These combined effects can slow neurodegeneration and improve behavioral symptoms. Nevertheless, the specific mechanisms linking exercise to circadian regulation in AD remain insufficiently understood, and further research is needed to clarify how these interactions contribute to therapeutic outcomes.

## 4 Summary and Future Perspectives

Over the past several years, physical exercise has emerged as an important behavioral regulator of circadian function. It supports daily rhythms, reinforces their amplitude, and promotes synchronization between central and peripheral oscillators. Through molecular, metabolic, immune, and neurovascular mechanisms, exercise contributes to improved sleep quality, metabolic efficiency, and brain resilience. These effects may be relevant in neurological diseases, where circadian disruption is increasingly recognized as a factor contributing to disease progression and impaired recovery. Despite growing evidence, however, several important questions remain unresolved. The effects of exercise on circadian rhythms may vary among individuals according to age, sex, chronotype, and disease state, but the factors underlying these differences are not yet fully understood. Future studies should clarify how exercise timing and disease stage influence circadian regulation and clinical outcomes. In addition, emerging evidence suggests that exercise should be considered not only as a lifestyle intervention, but also as a factor that influences temporal organization throughout the body. The benefits of exercise may depend not only on the amount of exercise performed, but also on its timing relative to an individual's circadian phase and disease status. This concept raises the possibility that exercise may serve as a practical form of behavioral chronotherapy in neurological disorders. If this is true, reliable methods for assessing circadian status and responsiveness will be required. One important challenge is therefore the development of reliable biomarkers of circadian resilience. Current measures often focus on individual circadian outputs and may not fully reflect the ability of the circadian system to adapt to physiological stress or disease. Improved biomarkers could facilitate personalized exercise prescriptions and help identify individuals most likely to benefit from circadian‐based interventions. Ultimately, further integration of circadian biology, exercise physiology, and clinical neurology may provide new opportunities for developing targeted interventions. Defining the optimal timing, intensity, and duration of exercise for specific disease contexts may contribute to individualized approaches for disorders associated with circadian disruption. Exercise should therefore be regarded not only as a lifestyle intervention but also as a potential strategy for maintaining circadian homeostasis and supporting long‐term neurological health.

## Funding

This study was supported in part by the National Institutes of Health (R01NS113556), grant from the Leducq Foundation, and a fellowship from the Takeda Science Foundation.

## Conflicts of Interest

Ken Arai is the Associate Editor of CNS Neuroscience and Therapeutics and a co‐author of this article. He is excluded from editorial decision‐making related to the acceptance and publication of this article. Editorial decision‐making was handled independently by the other editor to minimize bias.

## Data Availability

Data sharing not applicable to this article as no datasets were generated or analysed during the current study.
